# Cyberchondria, Fear of COVID-19, and Risk Perception Mediate the Association between Problematic Social Media Use and Intention to Get a COVID-19 Vaccine

**DOI:** 10.3390/vaccines10010122

**Published:** 2022-01-14

**Authors:** Daniel Kwasi Ahorsu, Chung-Ying Lin, Zainab Alimoradi, Mark D. Griffiths, Hsin-Pao Chen, Anders Broström, Toomas Timpka, Amir H. Pakpour

**Affiliations:** 1 Department of Rehabilitation Sciences, The Hong Kong Polytechnic University, Hung Hom 999077, Hong Kong; daniel.ahorsu@connect.polyu.hk; 2 Institute of Allied Health Sciences, College of Medicine, National Cheng Kung University, Tainan 701, Taiwan; cylin36933@gmail.com; 3 Social Determinants of Health Research Center, Research Institute for Prevention of Non-Communicable Diseases, Qazvin University of Medical Sciences, Qazvin 3419759811, Iran; zainabalimoradi@yahoo.com; 4 Psychology Department, Nottingham Trent University, Nottingham NG1 4FQ, UK; mark.griffiths@ntu.ac.uk; 5 Division of Colon and Rectal Surgery, Department of Surgery, E-DA Hospital, Kaohsiung 824, Taiwan; 6 School of Medicine, College of Medicine, I-Shou University, Kaohsiung 824, Taiwan; 7 Department of Nursing, School of Health and Welfare, Jönköping University, 553 33 Jönköping, Sweden; anders.brostrom@ju.se; 8 Department of Health, Medicine, and Caring Sciences, Linköping University, 581 83 Linköping, Sweden; toomas.timpka@liu.se

**Keywords:** vaccination, COVID-19, cyberchondria, fear of COVID-19, risk perception, problematic social media use, intention to get a COVID-19 vaccine

## Abstract

Vaccination is the most effective way to control the COVID-19 pandemic, but vaccination hesitancy threatens this effort worldwide. Consequently, there is a need to understand what influences individuals’ intention to get a COVID-19 vaccine. Restriction of information gathering on societal developments to social media may influence attitudes towards COVID-19 vaccination through exposure to disinformation and imbalanced arguments. The present study examined the association between problematic social media use and intention to get the COVID-19 vaccine, taking into account the mediating roles of cyberchondria, fear of COVID-19, and COVID-19 risk perception. In a cross-sectional survey study, a total of 10,843 residents of Qazvin City, Iran completed measures on problematic social media use, fear of COVID-19, cyberchondria, COVID-19 risk perception, and intention to get a COVID-19 vaccine. The data were analyzed using structural equation modeling (SEM). The results showed that there was no direct association between problematic social media use and intention to get a COVID-19 vaccine. Nonetheless, cyberchondria, fear of COVID-19, and COVID-19 risk perception (each or serially) mediated associations between problematic social media use and intention to get a COVID-19 vaccine. These results add to the understanding of the role of problematic social media use in COVID-19 vaccine hesitancy, i.e., it is not the quantity of social media use *per se* that matters. This knowledge of the mediating roles of cyberchondria, fear of COVID-19, and COVID-19 risk perception can be used by public health experts and policymakers when planning educational interventions and other initiatives in COVID-19 vaccination programs.

## 1. Introduction

The novel coronavirus disease 2019 (COVID-19) continues to spread around the world, although the rate is decreasing. By December 2021, there had been over 264 million confirmed cases and over 5.25 million deaths worldwide [1]. In Iran (where the present study was carried out), there have been over 6.12 million confirmed cases and over 130,000 deaths by December 2021 [1]. Vaccination is the most effective way to control the COVID-19 pandemic, and since COVID-19 was first identified in late 2019, the pace at which vaccines have been developed and distributed is unprecedented [2,3]. Nonetheless, one year after these vaccines became available, the number of vaccinated individuals is still far from a protective level, even in regions with high vaccine availability [4]. By late November 2021, approximately 4.25 billion individuals out of a total population of 7.9 billion were vaccinated worldwide (3.35 billion fully and 899.67 million partly vaccinated) and 56.65 million out of a total 85.5 million Iranians were vaccinated (44.73 and 11.93 million fully and partly, respectively) [5,6].

These numbers suggest that official health communications have not been accepted by large population factions [7,8]. Studies have reported that COVID-19 has disproportionately affected racial/ethnic minority groups and those who are economically and socially disadvantaged [9,10,11,12]. Therefore, achieving not just vaccine equality (i.e., similar allocation of vaccine supply proportional to its population across jurisdictions) but equity (i.e., preferential access and administration to those who have been most affected by COVID-19 disease) is an important goal [13]. Particular attention needs to be given to vaccination hesitancy, which may influence the possibility to achieve population immunity, especially among communities heavily exposed to social media [14,15]. It has been reported that sex (females), age (younger adults), educational level (fewer years of education), and socioeconomic status (family size and neighborhood) are independently and positively associated with the intention not to be vaccinated (i.e., with vaccine resistance or hesitancy) [10,11,16,17].

Problematic social media use is where an individual exhibits excessive social media use such that it affects other important areas of functioning, such as education/occupation, relationship, and/or quality of life [18,19,20]. This may start out of necessity or fun but may prove difficult to reduce or stop at a later time. Moreover, the use of social media for communication (e.g., contacting family members, friends, and business partners), pleasure (e.g., watching videos, movies, and gambling), and/or information purposes became a necessity for millions of people during the COVID-19 lockdown [21,22,23,24]. Therefore, prolonged exposure or being overly dependent on social media for information may lead to problematic social media use (and social media addiction in its most extreme form). This behavior has been associated with other mental health conditions [21,25,26] and also been found to influence fear of COVID-19 [21,27], cyberchondria [27], and COVID-19 risk perception [28]. It has also been posited that these mental health conditions, especially anxiety, are not only due to the consequence of the pandemic itself but also due to long-term isolation, changes in the structuring of time, and lifestyle [29]

Fear of COVID-19 has been reported to be positively associated with problematic social media use [27]. This is understandable because social media is used as one of the main sources for acquiring information concerning COVID-19. Therefore, the higher the fear of COVID-19, the more some individuals will strive to fully understand the disease, which will result in more time spent on social media. Apart from acquiring information, social media is also used to communicate with friends and relatives, which may help to allay an individual’s fear of COVID-19, especially because physical contacts are limited. Therefore, it can be speculated that, on one hand, social media use may help to allay the fear of COVID-19, and therefore the tendency to overuse social media increases during lockdowns due to a decrease in physical contact with family and/or friends. However, on the other hand, problematic use of social media may not relate well with fear of COVID-19. That is, problematic social media use may increase fear of COVID-19 [27]. It has also been reported that an association between fear of COVID-19 and COVID-19 risk perception exists where higher levels of fear of COVID-19 relate to higher levels of COVID-19 risk perception [30].

Cyberchondria, a behavior characterized by excessive and repeated online searching for health-related information that is driven by increasing levels of health anxiety, involves both an abnormal behavioral pattern and a heightened emotional state [31,32]. Therefore, higher levels of health anxiety, obsessive-compulsive symptoms, neuroticism, somatic symptoms, and intolerance of uncertainty are all associated with cyberchondria severity [33,34,35]. Consequently, individuals with such symptoms may be prone to severe cyberchondria unless they have psychologically protective factors such as optimism and resilience in coping with cyberchondria [34]. Similar to hypochondriasis (a condition characterized by a prolonged obsession with a strong fear of having a serious disease), cyberchondria thrives on the fear and anxiety over a disease [27,31,32,36,37], although it is yet to be a separate diagnosis in the *Diagnostic and Statistical Manual of Mental Disorders* [36]. Therefore, it is not surprising that several studies have reported a positive association between cyberchondria and fear of COVID-19 [27,38]. This indicates that the higher the level of cyberchondria, the higher the level of fear of COVID-19. With respect to the present study (on intention to get the COVID-19 vaccine), cyberchondria is uniquely different from problematic social media use. More specifically, in relation to COVID-19, cyberchondria comprises the search for online information on COVID-19 and the intention to get the COVID-19 vaccine, while problematic social media use comprises individuals being overly dependent on social media for a wide range of activities such as chatting, posting, and sharing information with others, some of which may include information on COVID-19 and the intention to get a COVID-19 vaccine. However, both cyberchondria and problematic social media use depend on the internet, and both may result in psychological symptoms such as anxiety.

Knowledge concerning the associations between problematic social media use, fear of COVID-19, cyberchondria, and COVID-19 risk perception and the intention to get a COVID-19 vaccine is important when planning COVID-19 vaccination programs. Therefore, the present study examined the association between problematic social media use and the intention to get a COVID-19 vaccine. It was hypothesized that cyberchondria, fear of COVID-19, and COVID-19 risk perception would be potential mediators of the aforementioned association.

## 2. Materials and Methods

### 2.1. Participants and Procedure

The present study was conducted in Qazvin City, Iran from February to April 2021. Using multi-stage cluster sampling, Qazvin city was divided into 70 strata. In each stratum, there were health centers that had a list of families of members in their catchment areas. In each stratum, health centers were randomly selected, and in each center, households were randomly selected from the family list and were then contacted by telephone. After explaining the objectives of the study in detail, 20 trained interviewers interviewed those who were willing to participate. Consequently, each trained interviewer collected data from approximately 540 interviewees (participants) each. The participation rate was 78% and there was no significant difference between participants and those who did not participate. The inclusion criteria included being over 18 years of age and the ability to read and write in Persian. The study design was approved by the Ethics Committee of Qazvin University of Medical Sciences (IR.QUMS.REC.1399.418). All participants provided their written informed consent before participation in the study.

### 2.2. Measures

#### 2.2.1. Endpoint Measure

Vaccination intentionality: The Intention to Get a COVID-19 Vaccination Scale (ICVVS) is a two-item scale (sample item: *“I am willing to get a COVID-19 vaccination”*) used to assess intention to get a COVID-19 vaccination. The items of the scale are rated on a five-point Likert scale from 1 (*strongly disagree*) to 5 (*strongly agree*). The items are summed together to obtain a total score, with a higher score indicating a higher level of intention to get a COVID-19 vaccination. The Persian ICVVS has satisfactory psychometric properties [30]. The psychometric properties for this study were 0.92 for Cronbach’s alpha (α), 0.91 for composite reliability (CR), and 0.84 for average variance extracted (AVE).

#### 2.2.2. Explanatory Factor

Problematic social media use: The Bergen Social Media Addiction Scale (BSMAS) is a six-item scale (sample item: *“How often during the last year have you felt an urge to use social media more and more?”*) used to assess problematic social media use [39]. The items of the scale are rated on a five-point Likert scale from 1 (*very rarely*) to 5 (*very often*). All the items are summed together to obtain a total score, with a higher score indicating a higher level of problematic social media use. In addition, a score of over 19 indicates an individual exhibiting problematic social media use [40]. The Persian BSMAS has satisfactory psychometric properties [41]. The psychometric properties for this study were 0.86 for Cronbach’s alpha (α), 0.85 for composite reliability (CR), and 0.44 for average variance extracted (AVE).

#### 2.2.3. Mediating Factors

Fear of COVID-19: The Fear of COVID-19 (FCV-19S) is a seven-item scale (sample item: *“I am most afraid of coronavirus-19”*) that is used to assess fear of COVID-19. The items of the scale are rated on a five-point Likert scale from 1 (*strongly disagree*) to 5 (*strongly agree*). All the items are summed together to obtain a total score, with a higher score indicating a higher level of fear of COVID-19. Previous studies have indicated that this scale has satisfactory psychometric properties in different language versions, including the Persian version [30,42,43]. The psychometric properties for this study were 0.88 for Cronbach’s alpha (α), 0.87 for composite reliability (CR), and 0.48 for average variance extracted (AVE).

Cyberchondria: The Cyberchondria Severity Scale–Short Form (CSS-12) is a 12-item scale (sample item: *“If I notice an unexplained bodily sensation I will search for it on the internet”*) used to assess cyberchondria behaviors [44]. The items of the scale are rated on a five-point Likert scale from 1 (*never*) to 5 (*always*). All the items are summed together to obtain a total score, with a higher score indicating a higher level of cyberchondria. The Persian CSS-12 has satisfactory psychometric properties [27]. The psychometric properties for this study were 0.89 for Cronbach’s alpha (α), 0.86 for composite reliability (CR), and 0.45 for average variance extracted (AVE).

COVID-19 risk perception: The Scale for Risk Perception (SRP) is a two-item scale (sample item: *“How likely do you think it is that you will be directly and personally affected by COVID-19 in the next 6 months?”*) used to assess how participants perceive their chance of getting COVID-19. The items of the scale are rated on a five-point Likert scale from 1 (*not at all*) to 5 (*very likely*). All items are summed together to obtain a total score, with a higher score indicating a higher level of risk perception. The SRP had satisfactory reliability in the present study (0.84). The psychometric properties for this study were 0.90 for Cronbach’s alpha (α), 0.87 for composite reliability (CR), and 0.50 for average variance extracted (AVE).

### 2.3. Data Analysis

Firstly, data analysis was carried out to present demographic characteristics of the participants using frequencies (*n*), means (M), and standard deviations (SD). Secondly, the Pearson correlation was used to examine the relationships between the variables used in this study. Thirdly, a model reflecting the present study’s proposed model was evaluated using structural equation modeling (SEM) along with full information maximum likelihood estimation (FIML). Then, fit indices were checked to ascertain that they suggested a good data-model fit before scrutinizing path coefficients in the SEM model. More specifically, the Tucker–Lewis index (TLI) together with the comparative fit index (CFI) >0.9 and the standardized root mean square residual (SRMR) together with the root mean square error of approximation (RMSEA) <0.08 were used as limits to determine whether data fitted well with the proposed model [45,46,47]. Given that the problem of over-sensitivity to a large sample size for the chi-square test (such as the present study having more than 10,000 participants), the non-significant chi-square test was not used for determining the data-model fit [48]. Additionally, a bootstrapping method with 10,000 resamples was applied to examine whether the proposed model contained any significantly direct or indirect effects. The bootstrapping method was conducted and presented using the bias-corrected bootstrapped confidence intervals (CIs) [49]. Fourthly, in order to provide practical information regarding how risk perception, fear of COVID-19, problematic social media use, and cyberchondria predicted a participant’s intention to get COVID-19 vaccine, a logistic regression was constructed. In the logistic regression, intention to get COVID-19 vaccine (ICVVS scored <5 as the reference group of not willing to get vaccinated, and scored 5 and above as the comparison group of willing to get vaccinated), and problematic social media use (BSMAS scored <19 as no problematic social media use, and scored 19 and above as problematic social media use) were dichotomized; the other variables (including risk perception, fear of COVID-19, and cyberchondria) were treated as continuous variables in the logistic model. All statistical analyses were performed using the IBM SPSS 24.0 or IBM AMOS 24.0 (IBM SPSS, Chicago, IL, USA).

## 3. Results

A total of 10,843 participants (4092 males; 37.7%) with a mean age of 35.54 years (SD = 12) successfully responded to all the measures in the present study (see Table 1). Most of these participants had a university degree (*n* = 4230, 39%) with few having no formal education (*n* = 352, 3.2%). Additionally, the majority of the participants were married (*n* = 8092, 74.6%) and lived in an urban area (*n* = 8186, 75.5%).

Table 2 shows the unadjusted correlations between the variables (fear of COVID-19, problematic social media use, risk perception, cyberchondria, and intent to get a COVID-19 vaccination) used in this study. It can be observed that there were significant positive correlations between all the study’s variables, with magnitudes ranging from 0.275 to 0.430 (all *p*-values < 0.01).

**Figure 1:**
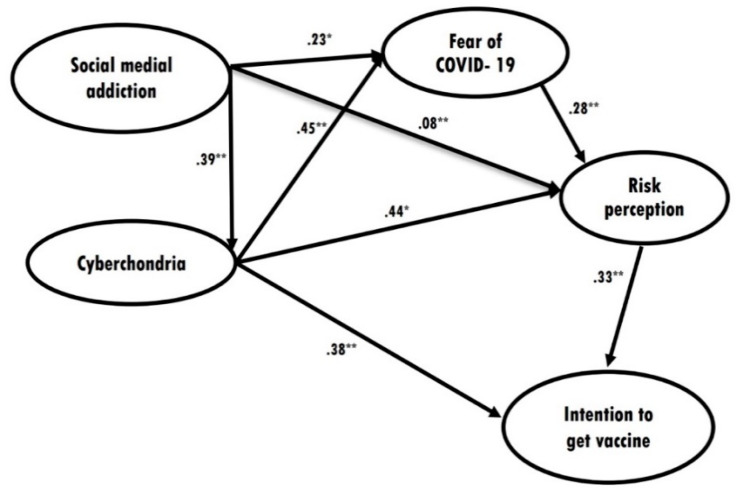
Final structural model of intention to get a COVID-19 vaccine adjusted for age, sex, marital status, education, and area of residence with standardized path coefficients displayed (χ^2^ (df) = 2081.479 (349); *p* < 0.001). Comparative fit index = 0.969. Tucker–Lewis index = 0.956. RMSEA = 0.060 (0.057–0.062). Standardized root–mean–square residual = 0.0574. * *p* < 0.05. ** *p* < 0.01.

Table 3 shows the direct, indirect, and total standard effects of the statistically significant associations between the variables included in the path analysis. No statistically significant association between problematic social media use and intention to get a COVID-19 vaccine was observed in the analysis. Figure 1 shows that the association between problematic social media use and intention to get a COVID-19 vaccine was mediated by cyberchondria and COVID-19 risk perception. Furthermore, problematic social media use conveyed an indirect effect through fear of COVID-19 and COVID-19 risk perception (*β* = 0.281; 95% CI = 0.242., 0.335). Likewise, cyberchondria conveyed an indirect effect on intention to get a COVID-19 vaccine through COVID-19 risk perception. All indirect effects were significant (*p* < 0.001).

Table 4 shows the results of a logistic regression analysis examining the effects of fear of COVID-19, problematic social media use, risk perception, and cyberchondria on the likelihood of intention to get a COVID-19 vaccine. More specifically, risk perception (aOR = 1.162, 95% CI = 1.078–1.253), fear of COVID-19 (aOR = 1.081, 95% CI = 1.051–1.111), problematic social media use (aOR = 1.122, 95% CI = 1.094–1.150), and cyberchondria (aOR = 1.049, 95% CI = 1.031–1.067) were all significantly associated with increased likelihood of participants getting a COVID-19 vaccine.

## 4. Discussion

The present study examined the association between problematic social media use and intention to get a COVID-19 vaccine, taking into account the mediating roles of cyberchondria, fear of COVID-19, and COVID-19 risk perception. The analysis found no direct association between problematic social media use and intention to get a COVID-19 vaccine, but a direct positive association between problematic social media use and fear of COVID-19, problematic social media use and cyberchondria, and problematic social media use and COVID-19 risk perception. These results indicate that problematic social media use may lead to an increase in other predisposition factors, but that problematic social media use *per se* does not influence the intention to get a COVID-19 vaccine. In other words, there is no direct association between problematic social media use and individual intention to get a COVID-19 vaccine.

The study also found direct positive associations between cyberchondria and fear of COVID-19, COVID-19 risk perception, and intention to get a COVID-19 vaccine. These findings support previous studies that have reported a positive association between cyberchondria and fear of COVID-19 [27,38]. It is worth noting that the present study added new findings—positive associations between cyberchondria and COVID-19 risk perception and between cyberchondria and intention to get a COVID-19 vaccine. Furthermore, the study found a direct association between fear of COVID-19 and COVID-19 risk perception, but not between fear of COVID-19 and intention to get a COVID-19 vaccine. Therefore, it can be inferred that fear of COVID-19 and intention to get a COVID-19 vaccine are associated via a mediator. This echoes a previous study which also found a direct association between fear of COVID-19 and COVID-19 risk perception, but not between fear of COVID-19 and intention to get a COVID-19 vaccine [30]. Additionally, there was a direct association between COVID-19 risk perception and intention to get a COVID-19 vaccine, which indicates that an individual’s own estimate of risk is what constitutes the strongest influence on one’s intention to get a COVID-19 vaccine [30].

It was also found that increased risk perception, fear of COVID-19, cyberchondria, and problematic social media use were associated with the increased likelihood of participants getting a COVID-19 vaccine. These findings indicate that higher levels of these factors may help increase an individual’s intention to get a COVID-19 vaccine. This is supported by studies that reported that more individuals were willing to get COVID-19 vaccination during the lockdown compared to the pre-lockdown period of COVID-19 and, as risk perception increased, so did the intention to accept getting the vaccine [50,51,52]. Social media plays a significant role in COVID-19 vaccination uptake [53,54]. Therefore, it is not surprising that having problematic social media use and cyberchondria are associated with the increased likelihood of participants getting a COVID-19 vaccine. The important issue here will be the kinds of information that individuals are accessing, and not the behavior itself. Consequently, health experts and communicators should be proactive in putting out factual information on COVID-19, while media (including social media) regulators should oversee the media landscape so as to eradicate all fake news.

### 4.1. Implications for Vaccination Practice

The study’s findings add novel evidence for COVID-19 vaccination practice. The observation that there was no direct association between problematic social media use and intention to get a COVID-19 vaccine implies that there is no reason to recommend limiting social media use on the whole. However, although high levels of social media use do not directly influence an individual’s intention to get a COVID-19 vaccine, it may be influenced through factors such as cyberchondria, COVID-19 risk perception, and fear of COVID-19 and COVID-19 risk perception (serially). This implies that it is not the quantity, but the quality of social media use (i.e., the content that is accessed) that influences vaccination intent. Cyberchondria was indirectly associated with the intention to get a COVID-19 vaccine via (i) COVID-19 risk perception and (ii) fear of COVID-19 and COVID-19 risk perception. These findings are novel contributions from the present study. In addition, fear of COVID-19 was indirectly associated with the intention to get a COVID-19 vaccine via risk perception, although there was no direct association between fear of COVID-19 and intention to get a COVID-19 vaccine. This suggests that fear of COVID-19 may not be sufficient to influence an individual’s intention to get a COVID-19 vaccine, but that the individual’s COVID-19 risk perception also has to be taken into account. This finding confirms a previous study that reported an association between these variables [30]. In addition, the increase in these variables is associated with the increased likelihood of participants getting a COVID-19 vaccine, which re-emphasizes the need for factual information on COVID-19 and the elimination of fake news from the media landscape. Hence, health authorities and experts/communicators and media regulators should work in tandem to provide correct information on COVID-19.

### 4.2. Strengths and Limitations

The present study has several limitations. First, although a large population sample was recruited, it should be taken into consideration that convenience sampling was employed, which limits the representativeness of the study population. Therefore, caution should be exercised when generalizing the findings, taking into account population age, educational level, religion, and social media access possibilities. Second, a cross-sectional design was used, which means that causal associations between the variables studied cannot be determined. Third, all the data were collected using self-report measures, which are subject to biases such as social desirability. However, the psychometric properties for all the self-report instruments are robust, suggesting validity and trustworthiness of the data to an appreciable degree. Fourth, there is a possible concept overlap between cyberchondria and problematic social media use, and so readers should be cautious in overly emphasizing the uniqueness of cyberchondria and problematic social media use concerning their roles in getting the COVID-19 vaccine.

## 5. Conclusions

The present study found no direct association between problematic social media use and the intention to get a COVID-19 vaccine, but did find several indirect associations via cyberchondria, fear of COVID-19, and COVID-19 risk perception (each or in combination) as mediators. This implies that it is not the quantity of social media use but rather the quality of the content that has the most influence on vaccination intent. Additionally, given the nascent research, the term ‘cyberchondria’ should be approached with caution and viewed as a preliminary diagnostic proposal needing further empirical exploration. The study’s findings can be used by health experts, communicators, and policymakers when planning educational interventions such as interventional mapping and other initiatives in COVID-19 vaccination programs that may increase risk perception and/or decrease cyberchondria [55,56,57]. Future studies are needed to ascertain the role of other factors (e.g., religion) in acceptance of COVID-19 vaccination.

## Figures and Tables

**Table 1:** The demographic characteristics of the participants (*N* = 10,843).

	Mean (SD) or N (%)
*Age (years)*	35.54 (±12.00)
*Sex*	
Male	4092 (37.7%)
*Educational status*	
University	4230 (39.0%)
Diploma	2761 (25.5%)
High school	974 (9.0%)
Secondary school	1540 (14.2%)
Primary school	986 (9.1%)
No formal education	352 (3.2%)
*Marital status*	
Married	8092 (74.6%)
Single	2751 (25.4%)
*Area of residence*	
Urban	8186 (75.5%)
Rural	2656 (24.5%)

**Table 2:** Correlations between study variables.

	1	2	3	4	5	Mean	SD
1. Fear of COVID-19	1	0.257 **	0.421 **	0.533 **	0.422 **	21.12	6.94
2. Problematic social media addiction use	-	1	0.270 **	0.305 **	0.387 **	17.61	5.67
3. COVID-19 risk perception	-	-	1	0.410 **	0.398 **	3.76	1.89
4. Cyberchondria	-	-	-	1	0.430 **	29.13	8.91
5. COVID-19 vaccination intent	-	-	-	-	1	3.84	1.10

** *p* < 0.01.

**Table 3:** Direct, indirect, and total standard effects of the statistically significant associations between the study variables. The analyses were adjusted for age, sex, marital status, education, and area of residence.

Parameter	Total Effect (*p*-Value)	Direct Effect (*p*-Value)	Indirect Effect (*p*-Value)	Bootstrapping SE (LLCI, ULCI)
Problematic social media use → Fear of COVID-19	0.401 (0.009)	0.225 (0.021)	0.176 (0.003)	0.018 (0.146, 0.221)
Problematic social media use → Cyberchondria	0.390 (0.006)	0.390 (0.006)	-	-
Problematic social media use → COVID-19 risk perception	0.361 (0.004)	0.080 (0.008)	0.281 (0.004)	0.021 (0.242, 0.335)
Cyberchondria → Fear of COVID-19	0.451 (0.005)	0.451 (0.005)	-	-
Cyberchondria → COVID-19 risk perception	0.561 (0.009)	0.437 (0.018)	0.124 (0.003)	0.021 (0.91, 0.180)
Cyberchondria → Intention to get a COVID-19 vaccine	0.566 (0.006)	0.379 (0.007)	0.187 (0.003)	0.022 (0.148, 0.247)
Fear of COVID-19 → COVID-19 risk perception	0.275 (0.004)	0.275 (0.004)	-	-
COVID-19 risk perception → Intention to get a COVID-19 vaccine	0.333 (0.005)	0.333 (0.005)	-	-

**Table 4:** Results of logistic regression model in explaining intention to get a COVID-19 vaccine.

		95% CI	
Variable	aOR	Lower	Upper
COVID-19 risk perception	1.162	1.078	1.253
Fear of COVID-19	1.081	1.051	1.111
Problematic social media use	1.122	1.094	1.150
Cyberchondria	1.049	1.031	1.067

aOR = adjusted odds ratio; CI = confidence interval.

## Data Availability

Data are available on reasonable request from the corresponding author.
